# Anticancer, antioxidant, antiviral and antimicrobial activities of Kei Apple (*Dovyalis caffra*) fruit

**DOI:** 10.1038/s41598-022-09993-1

**Published:** 2022-04-08

**Authors:** Husam Qanash, Reham Yahya, Marwah M. Bakri, Abdulrahman S. Bazaid, Sultan Qanash, Abdullah F. Shater, Abdelghany T. M.

**Affiliations:** 1 Department of Medical Laboratory Science, College of Applied Medical Sciences, University of Ha’il, PO Box 2440, Hail, Saudi Arabia; 2 Molecular Diagnostics and Personalized Therapeutics Unit, University of Ha’il, PO Box 2440, Hail, Saudi Arabia; 3 College of Science and Health Professions, King Saud Bin Abdulaziz University for Health Sciences, Riyadh, Saudi Arabia; 4 King Abdullah International Medical Research Center, Riyadh, Saudi Arabia; 5 Biology Department, Faculty of Science, Jazan University, Jazan, Saudi Arabia; 6 Medicine Department, Ministry of the National Guard-Health Affairs, Jeddah, Saudi Arabia; 7 King Abdullah International Medical Research Center, Jeddah, Saudi Arabia; 8 King Saud Bin Abdulaziz University for Health Sciences, Jeddah, Saudi Arabia; 9 Department of Medical Laboratory Technology, Faculty of Applied Medical Sciences, University of Tabuk, Tabuk, Saudi Arabia; 10 Botany and Microbiology Department, Faculty of Science, Al-Azhar University, Cairo, Egypt

**Keywords:** Biochemistry, Biophysics, Cell biology, Microbiology

## Abstract

Secondary plant metabolites remain one of the key sources of therapeutic agents despite the development of new approaches for the discovery of medicinal drugs. In the current study, chemical analysis, and biological activities of Kei apple (*Dovyalis caffra*) methanolic extract were evaluated. Chemical analysis was performed using HPLC and GC–MS. Antiviral and anticancer effect were assessed using the crystal violet technique and activity against human liver cells (HepG2), respectively. Antibacterial activity was tested with the disc diffusion method. The obtained results showed that chlorogenic acid (2107.96 ± 0.07 µg/g), catechin (168 ± 0.58 µg/g), and gallic acid (15.66 ± 0.02 µg/g) were the main bioactive compounds identified by HPLC techniques. While, compounds containing furan moieties, as well as levoglucosenone, isochiapin B, dotriacontane, 7-nonynoic acid and tert-hexadecanethiol, with different biological activities were identified by GC–MS. Additionally, inhibition of 2,2-diphenyl-1-picryl-hydrazyl-hydrate (DPPH) scavenging was 79.25% at 2000 µg/mL, indicating its antioxidant activity with IC_50_ of 728.20 ± 1.04 µg/mL. The tested extract exhibited potential anticancer activity (58.90% toxicity) against HepG2 cells at 1000 µg/mL. Potential bacterial inhibition was observed mainly against *Escherichia coli* and *Proteus vulgaris*, followed by *Staphylococcus aureus* and *Bacillus subtilis* with a diameter of growth inhibition ranging from 13 to 24 mm. While weak activities were recorded for fungi *Candida albicans* (10 mm). The extract showed mild antiviral activity against human coronavirus 229E with a selective index (SI) of 10.4, but not against human H3N2 (SI of 0.67). The molecular docking study's energy ratings were in good promise with the experiment documents of antibacterial and antiviral activities. The findings suggest that *D. caffra* juice extract is a potential candidate for further experiments to assess its use as potential alternative therapeutic agent.

## Introduction

*Dovyalis caffra* belongs to the family Salicaceae and is commonly cultivated as a protector at forest edges in numerous regions of the world as well as a hedge plant in Egypt^1,2^. *D. caffra* is an ethnic fruit tree in Southern Africa and other countries, where its diverse varieties are planted in arid and semiarid areas^3–5^. The common name of *D. caffra* is Kei apple, which is derived from the southwest African Kei River. The fruit origin was associated with South Africa, but then transferred to other countries. At maturity, Kei apple produces golden fruits containing soft yellow juicy pulp with an active aroma and a sour taste. Traditionally, in some countries, Kei apple fruits can be consumed fresh or used as additives to jams or preserves due to their heteropolysaccharide, ascorbic, l-malic acid, tannin, phenolic acid, and flavonoid contents.

The side effects of chemotherapeutic drugs and the high cost of treatment represent the highest restrictions of conventional therapy and create a significant issue in the treatment of many diseases, including cancer^6^. Cancer is one of the main fatal diseases worldwide, leading to approximately 9.9 million deaths in 2020^7^. In addition, the emergence of infections caused by multidrug-resistant bacteria has worsened the situations^8^. Therefore, novel and safe therapeutic strategies are becoming a priority. Plants are potential alternatives that have activity to treat many diseases, such as infections, cancers, and antioxidant properties^9^. Plant extracts continue to play a main role in drug discovery and are promising sources for phenolic and flavonoid contents^10,11^. Various plant extracts, including *D. caffra,* have been proposed for their medical use^12^, although their biological activities in many plants have still been ambiguous. In a recent study, the nutritional and health prospective of *D. caffra* fruits were reported, but the fruit contents and its important in food industry and therapeutic field were still unexploited in numerous countries due to limited research and lack of scientific information, as well as absence of agro-processing techniques^2^.

Strong antioxidant activity of Kei apples fruit was previously reported due to the high content of phenolic, flavonoid, and amino acids^13^. In addition to that, A positive correlation was recorded between antioxidant and concentrations of polyphenols of *Dovyalis caffra* fruits^13^. A previous study also showed an antifungal activity of *D. caffra*-derived fruit juice against *Microsporum canis*, *Malassezia furfur* and *Candida albicans*^14^. Furthermore, antibacterial activities were observed against *Staphylococcus aureus*^4^. Even though these scientific reports have highlighted the antimicrobial activities of *D. caffra*, studies to evaluate the potential therapeutic use of *D. caffra* fruit would extensively be necessary. Therefore, the current research aimed to assess various in vitro biological activities of *D. caffra* fruit extracts, including antiviral, antitumor and antibacterial and antifungal activities, with phytochemical characterization.

## Material and methods

### Plant sample and extraction process

Ripe fruits (500 g) were collected during Augustus 2020 from trees of *D. caffra* cultivated from a farm in Egypt. Identification of the plant was performed according to Venter et al.^15^ with further authentication achieved by Taxonomist. The collected fruits were washed to remove any dust, using a stream of running tap water and then pulped (skin and flesh) in an electric mixer for further extraction. Through a 1 mm sieve, the pulp was filtered to obtain smooth pulp without skin or fiber. The pulp juice (250 mL) was extracted with 250 mL methanol, and then concentrated using a rotary evaporator to obtain the dried extract at 50 °C. A voucher sample of *D. caffra* material (DC4432) was deposited in herbal collection of plant.

### Chemical and reagents

All chemicals, reagents, solvents, buffers, and microbial growth media contents were obtained from Sigma-Aldrich, Saint Louis, MO, USA.

### Gas chromatography–mass spectrometry (GC/MS) analysis of *D. caffra* fruit extract

The content of the *D. caffra* extract was analyzed by a gas chromatography (Thermo Scientific Corp., USA) mass spectrometry (ISQ Single Quadrupole Mass Spectrometer) GC–MS. Separate capillary column TR-5MS (30 m × 0.32 mm × 0.25 μm film thickness) was applied for analysis at 60 °C as starting temperature, then raised up to 240 °C, followed by increasing by 30 °C/min up to maximum temperature 290 °C, which was isothermally continuous about two minutes. Temperature was adjusted at 250 °C and 260 °C to protect the injector and MS transfer, respectively. At constant flow, the applied carrier (helium) featured high purity at an amount of 1 mL/min. After three minutes, the solvent was cut, and the diluted fruit extract (1 µL) was inoculated with an AS1300 autosampler linked to GC in a split manner. Electron ionization mass spectra was collected in full scan mode in the range of m/z 40–1000 by electron energy of 70 eV application. The phytoconstituents of fruit extracts were identified and compared to the available information in library mass spectra at the National Institute of Standards and Technology (NIST) via calculation of their mass spectra and retention time (RT)^16^.

### High-performance liquid chromatography (HPLC) for flavonoid and phenolic content determination

HPLC (Series 1100, Agilent Technologies, USA) was used to detect flavonoids and phenolic acid compounds. Gradient was the mode of elution at run rate (1 mL/min). Wavelength monitoring was performed at 280 nm. The extracted fruit juice (50 mL) with 200 mL of methanol (80%) was filtered through a 0.22 µm syringe filter and injected (10 µL) into the HPLC (Column C18 Inertsil: 4.6 × 250 mm, 5 µm) with 0.1% phosphoric acid in water as a buffer and in methanol as mobile phase. Column temperature was set at 20 °C. Different standard stock solutions of phenolics and flavonoids in methanol were prepared and injected as mentioned for the fruit sample^17^.

### Antimicrobial Activity of plant extract

*D. caffra* fruit extract was tested against some bacteria and fungi via disc diffusion method assay; two Gr+ve (*Bacillus subtilis* NRRL B-543 and *Staphylococcus aureus* ATCC 25923), and two Gr-ve bacteria (*Proteus vulgaris* ATCC 13315 and *Escherichia coli* ATCC 25922) were used; filamentous fungus *Aspergillus fumigatus* RCMB 002008 and unicellular fungus *Candida albicans* ATCC 10231^18^ were provided by Regional Center for Mycology and Biotechnology (RCMB), Al-Azhar University, Cairo, Egypt.

Under sterile conditions, discs (5 mm) of filter paper (Whatman No. 1) were loaded with 20 µg/disc of the dried methanolic extract. The discs were left for 2 h to complete dryness, placed on prepared bacterial seeded nutrient agar and fungal seeded yeast extract peptone dextrose (YEPD) agar media, and finally kept for 30 min in a refrigerator for appropriate diffusion of the extract. Then, the plates were incubated for 24 h at 37 °C for bacteria, 2 days for yeast at 30 °C, and 5 days for fungus at 30 °C. The inhibition zone (mm) around discs was measured to record the antimicrobial activity of the fruit extract. Antibiotic (gentamycin) and antifungal (ketoconazole) were used as positive controls. Discs were loaded with methanol, as the extracted solvent of plant samples was also used as a control.

### Antiviral assay using Crystal Violet

The antiviral activity of the extract against human coronavirus 229E and H3N2 influenza was achieved by a cytopathogenic effect (CPE) inhibition assay that determined the antiviral effectiveness in cell culture systems. Human coronavirus HCoV 229E and Vero E6 cells from African green monkey kidney, and H3N2 virus and Madin-Darby canine kidney (MDCK) cells were provided by Nawah Scientific, Egypt. The cells were cultured in Dulbecco’s modified Eagle’s medium (DMEM) with 10% fetal bovine serum (FBS) and 0.1% antibiotic/antimycotic solution (Grand Island, New York, USA). Vero E6 and MDCK cells (2 × 10^4^ cells/well) were cultured in a 96-well plate, containing Dulbecco's Modified Eagle Medium (DMEM) for 24 h prior to infecting with human coronavirus 229E and H3N2 influenza virus, respectively. Then, the DMEM was removed, and the cells were washed with phosphate-buffered saline (PBS). The crystal violet technique was applied to assess antiviral activity and cytotoxicity assays for human coronavirus 229E and H1N1 virus infectivity, which monitored the virus-induced CPE and allowed the cell viability (%) to be calculated^19^. A diluted virus suspension (0.1 mL) of each virus with a 50% cell culture infective dose (CCID_50_) from the virus stock was inoculated in mammalian cells (this quantity was selected to show the desired CPEs). To detect the effect of the extract, 0.01 mL of medium supplemented with different concentrations of the extract (0.1–1000 µg/mL) was used as cultivable of the cells. Control cells (noninfected and nondrug-treated cells) and virus controls (virus-infected and nondrug-treated cells) were included in the experiment. The culture plates were incubated for 72 h at 37 °C in 5% CO_2_. The developed cytopathic effect was examined by light microscopy. After a washing step with PBS, the cell monolayers were fixed and stained with crystal violet solution (0.03%) in ethanol (2%) and formalin (10%). After that, the optical density of individual wells was measured at 540/630 nm utilizing a spectrophotometer. Antiviral activity (%) was designed as mentioned previously^20^, giving from the following equation:

$${\text{Antiviral}}\;{\text{activity}} = \frac{{{\text{ODCC}} - {\text{ODVC}}}}{{{\text{ODT}} - {\text{ODVC}}}} \times 100$$

where ODCC is a control optical density of cell, ODVC is a control optical density of virus, and ODT is a test optical density. The 50% CPE inhibitory concentration (IC_50_) was calculated based on the obtained results. The selective index (SI) was also estimated as CC50/IC50. Prior to this assay, cytotoxicity was assayed, and the cells (2 × 10^4^ cells/well) were seeded in a 96-well culture plate for 1 day. Then, medium containing different concentrations of the extract was added to the cells and incubated for 72 h before being detached, and the cells were washed with PBS. The next steps were approved in the same routine as designated for the assay of the antiviral activity above. Acyclovir as an antivirus was applied as a standard control.

### Antioxidant activity

2,2-Diphenyl-1-picryl-hydrazyl-hydrate (DPPH) free radical analysis was performed to determine the antioxidant activity of the extract. Briefly, the reaction included the addition of 100 µL of 0.1% DPPH reagent in methanol to 100 µL of the extract in a microplate (96 wells), followed by incubation at 25 °C in the dark for 30 min. The developed reduction in DPPH color reduction intensity was recorded via a microplate reader FluoStar Omega at 540 nm^21^. Estimation of the activity of DPPH radical scavenging (%) was recorded using the following formula:

$$\text{Activity\;of\;DPPH\;scavenging }(\%)= \frac{\text{Blank\;absorbance}-\text{Extract\;absorbance } }{\text{Blank\;absorbance}}\times 100$$

Fruit extracts were prepared to obtain final concentrations of 125–2000 µg/mL in DMSO to recognize a series inside which the inhibitory concentration 50% (IC_50_) lies. A stock solution of 40 µg/mL ascorbic acid as a standard was prepared in methanol at various dilutions (5, 10, 15, 20, 25, 30, 35 and 40 µg/mL).

### Antitumor assay

Human liver cancer (HepG2) was provided by Nawah Scientific Inc. in (Cairo, Egypt). HepG2 cells were cultured in DMEM supplemented with antibiotics (penicillin; 100 units/mL and streptomycin; 100 mg/mL) and 10% heat-inactivated fetal bovine serum (FBS) in a humidified 5% (v/v) CO_2_ atmosphere at 37 °C. HepG2 cell viability was detected via a sulforhodamine B (SRB) assay. Briefly, the cell suspension (100 μL) containing 5 × 10^3^ cells was cultured in 96-well plates containing medium treated with different levels of extract and incubated for 24 h. After exposing the cells to the treatments, the cells were fixed by substituting media with 150 μL of trichloroacetic acid (TCA) (10%) and preserved for 1 h at 4 °C, followed by TCA desiccant. After that, the cells were washed 5 times with distilled water. The solution of TCA was detached, and the cells were washed 4 times with distilled water. The cells were immersed in 70 μL of SRB solution (0.4% w/v) for 10 min in dark at 25 °C. Then, the plates were washed with 1% acetic acid 3 times and dried in air for 12 h. Dissolved protein-bound SRB staining was performed by the addition of 150 μL TRIS base solution (10 mM, pH 10.5), and the absorbance was measured at 540 nm using a BMG LABTECH^®^-FLUOstar Omega microplate reader (Ortenberg, Germany)^22^.

All experimental research and field studies were performed in accordance with the relevant international guidelines and regulations.

### Molecular docking of chlorogenic acid with HCoV-229E and *Proteus vulgaris*

Computational approaches that ‘dock’ small molecules into the structures of macromolecular targets and ‘score’ their potential complementarity to binding sites are widely used in hit identification and lead optimization. The structural model was built using the BUILDER module of MOE, Optimization Conformational analyses of the built molecules were performed in a two-step procedure. First, these compounds were submitted to energy minimization tool using the included MOPAC 7.0, the geometry of the compounds was optimized using the semiemperical PM3 Hamiltonian with Restricted Hartree–Fock (RHF) and RMS gradient of 0.05 kcal/mol. Then, the obtained model was implemented to the ‘Systematic Conformational Search’ of the MOE. To rank the binding affinity of the compounds to (6U7H) and (1HZO) proteins the binding free energy and hydrogen bonds between the compounds and amino acid in to (6U7H) and (1HZO) were used. Evaluation of the hydrogen bonds were done by measuring the hydrogen bond length, in addition, RMSD of the co-crystal ligand position compared to the docking pose was used in ranking. Both RMSD as well as the mode of interaction of the native ligands within Cryo-EM structure of the HCoV-229E spike glycoprotein (6U7H) and Structure of class A cephalosporinase from *Proteus vulgaris* K1 (1HZO) receptor were used as standard docked model.

### Statically analysis

The experiments were performed, and the data were calculated as the ± standard deviation means, using GraphPad Prism^®^ (version 5.0) software to obtain the IC_50_ value of DPPH radical scavenging activity graphs.

## Results and discussion

### Phytochemical characterization

**Figure 1:**
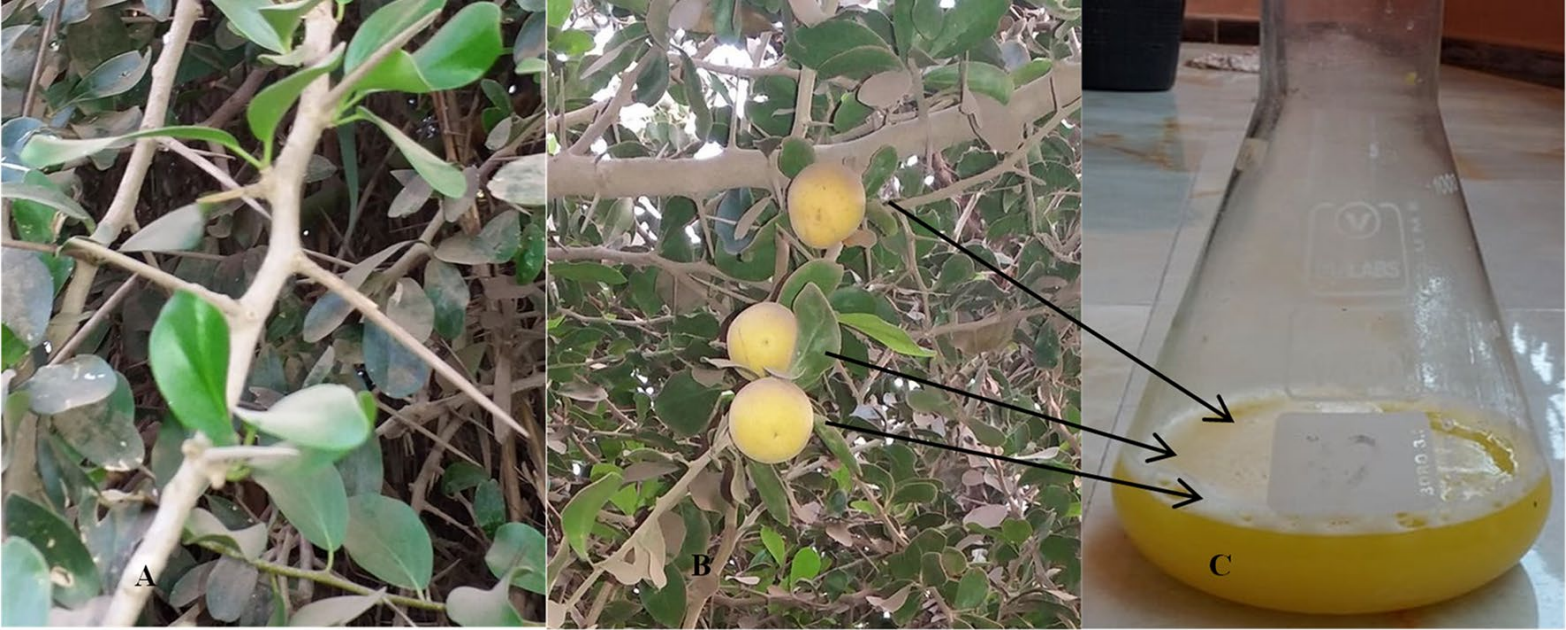
Different parts of *D. caffra* tree with (**A**) leaves, stem, and spines (**B**) Fruit ripe, and (**C**) its extract.

**Figure 2:**
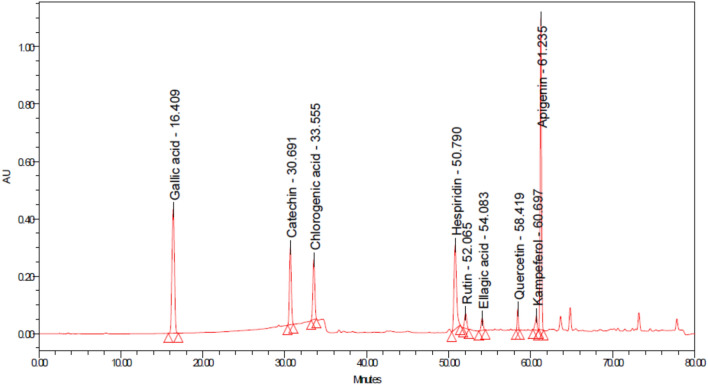
HPLC chromatogram detected of some phenolic and flavonoid contents of *D. caffra* fruit extract.

The extract was collected from yellow orange fruits at the ripe stage (Fig. 1). Kei apple fruits are hard and firm than their plum, and fruit color depends mainly on the ripeness of the raw materials^23^. The fruit produces extracts with a very acidic flavor that must be sweetened prior to ingestion. Phenolic and flavonoid content analysis by HPLC showed the existence of various compounds in Kei apple extract (Table 1 and Fig. 2). Chlorogenic acid was the main (2107.96 ± 0.07 µg/g) recognized phenolic compound in the extract, followed by catechin (168 ± 0.58 µg/g) and gallic acid (15.66 ± 0.02 µg/g) (Table 1). These results are in line with^13^, who reported the presence of chlorogenic acid at similar concentrations and found that chlorogenic acid was the predominant phenolic component of Kei apple fruit^13^. Chlorogenic acid was found in other fruits, such as pears, apples, vegetables, and green coffee beans^24^. Catechin was the second most abundant phenol of the whole fruit extract but not in the dried fruit^13^. Hydroxybenzoic acid (gallic acid) was also detected in the extract of the current study which is consistent with similar report where gallic acid was detected in Kei apple fruit extract at low concentrations^25^; however, gallic acid was not detected in some samples of Kei apple extracts^26^. Among the naturally detected products, apigenin and quercetin are important flavonoids, which exhibited potential anticancer activity^27,28^.

**Table 1:** Phenolic and flavonoid contents of *D. caffra* fruit extract.

Compound	Concentration (µg/g)
Gallic acid	15.66 ± 0.02
Catechin	168 ± 0.58
Chlorogenic acid	2107.96 ± 0.07
Hesperidin	2.10 ± 0.2
Rutin	4.45 ± 0.10
Ellagic acid	1.82 ± 0.10
Quercetin	2.13 ± 0.10
kaempferol	2.02 ± 0.02
Apigenin	1.72 ± 0.02

**Figure 3:**
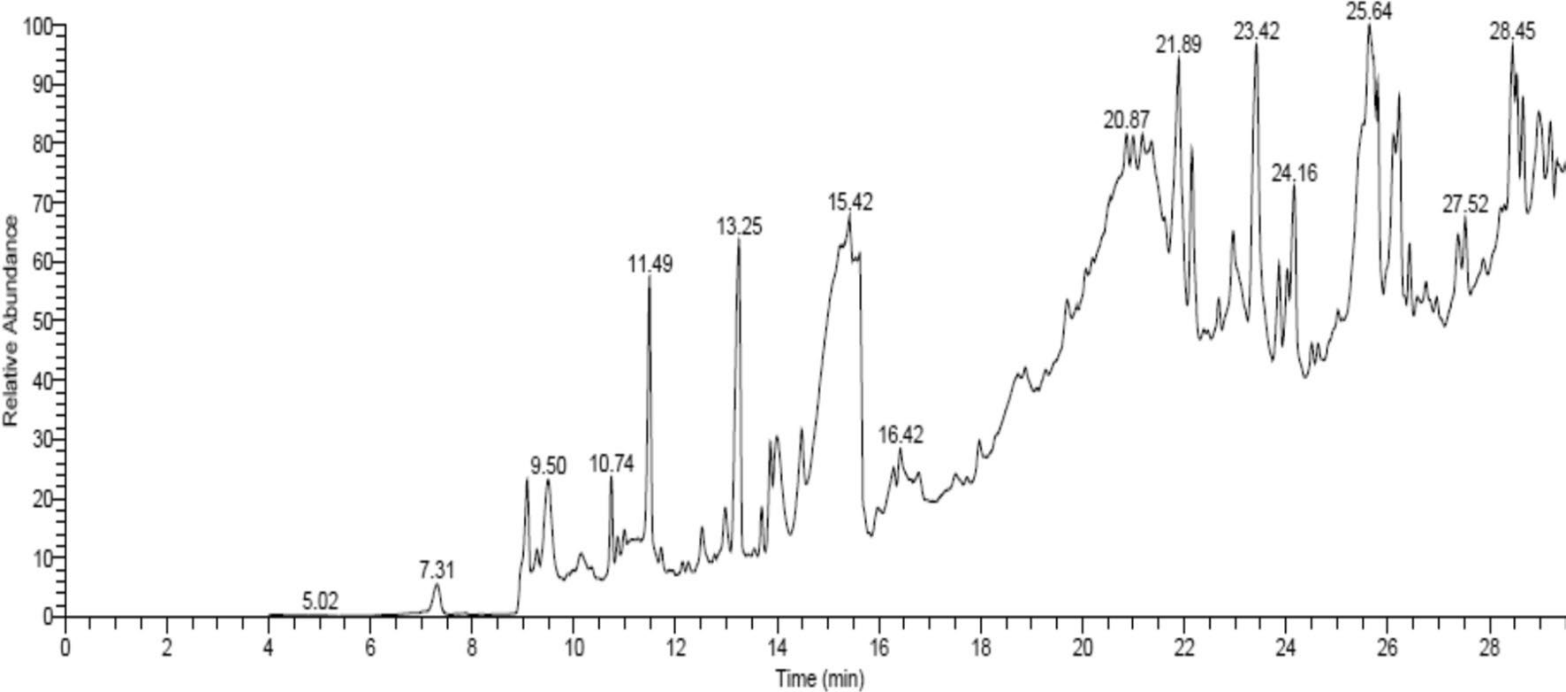
GC/MS chromatogram analysis of *D. caffra* fruits extract.

Further GC–MS analysis of the extract showed the presence of different compounds (Table 2 and Fig. 3), which possess some biological activities. Numerous compounds containing furan moieties are familiar structural styles in several products of natural origin, such as 3-Furaldehyde, 5-Methylfuran-2-Carbaldehyde, 2(5H)-Furanone, Furyl hydroxymethyl ketone and 2,4-Dihydroxy-2,5-dimethyl-3(2H)-furan-3-one. Anticancer activity was reported using methyl-5-(hydroxymethyl)-2-furan carboxylate and its derivatives against Vero cell HeLa and HepG2 lines^29^. Another study observed that the proliferation of bacteria was inhibited by furan compounds^30^. Levoglucosenone, sesquiterpene lactone (isochiapin B) and dotriacontane were recognized as constituents of Kei apple extract (Table 2), and their cytotoxic effects against hepatocarcinoma cell lines^31^, antioxidant activity^32^, and antibacterial and antiviral activities^33^ were documented.

**Table 2:** Phyto-constituents of *D. caffra* fruit extract identified by GC/MS.

Phyto-constituent	R.T.^a^	Area %	M.F.^b^	M.W.^c^
3-Furaldehyde	7.32	0.62	C_5_H_4_O_2_	96
Acetic acid	9.09	2.38	C_2_H_4_O_2_	60
á-Alanine	9.28	0.29	C_3_H_7_NO_2_	89
5-Methylfuran-2-Carbaldehyde	9.50	2.08	C_6_H_6_O_2_	110
Methyl 6-oxoheptanoate	10.15	0.45	C_8_H_14_O_3_	158
2(5H)-Furanone	10.74	1.16	C4H_4_O_2_	84
Hexadecanoic Acid	10.86	0.25	C16H_32_O_2_	256
7-Nonynoic acid	10.99	0.28	C9H_14_O_2_	154
2,4-Dihydroxy-2,5-dimethyl-3(2H)-furan-3-one	11.49	3.99	C_6_H_8_O_4_	144
1,5-Dinitroso-1,5-Diazocane	11.72	0.25	C_6_H_12_N_4_O_2_	172
4-Amino-1,5-pentandioic acid	12.52	0.75	C_7_H_13_NO_4_	175
Orcinol	12.98	0.95	C_7_H_8_O_2_	124
Furyl hydroxymethyl ketone	13.26	6.62	C_6_H_6_O_3_	126
Levoglucosenone	13.70	0.61	C_6_H_6_O_3_	126
Butanedioic acid, hydroxy-, dimethyl ester	13.87	1.39	C_6_H_10_O_5_	162
Methyl 2,4-Heptadienoate	14.48	1.29	C_8_H_12_O_2_	140
2-Butenedioic acid (E)-, monomethyl ester	15.09	2.76	C_5_H_6_O_4_	130
4-Methoxy-4-oxo-2-butenoic acid	15.21	2.62	C5H6O_4_	130
2-Butenedioic acid (Z)-, monomethyl ester	15.64	4.09	C5H6O_4_	130
5-Amino-1-benzoyl-1H-pyrazole-3,4-dicarbonitrile	15.97	0.41	C_12_H_7_N_5_O	237
Melezitose	16.30	0.51	C18H32O_16_	504
9,12-Octadecadienoyl chloride, (Z,Z)-	17.97	0.62	C_18_H_31_ClO	298
5-Hydroxymethylfurfural	18.74	0.68	C_6_H_6_O_3_	126
Cholestan-3-ol, 2-methylene-, (3á,5à)-	18.87	0.59	C_28_H_48_O	400
Tetradecanoic acid, 2-hydroxy	20.52	0.36	C_10_H_18_O_2_	170
Nerolidol-Epoxyacetate	21.00	0.60	C_17_H_28_O_4_	296
Pogostole	21.90	5.28	C_15_H_26_O	222
1-Isopropyl-4,8-dimethylspiro [4.5] dec-8-en-7-one	22.16	2.99	C_15_H_24_O	220
à-Kessyl acetate	22.68	0.54	C_17_H_28_O_3_	280
1-Heptatriacotanol	22.96	2.08	C_37_H_76_O	536
2-Aminoethanethiol hydrogen sulfate (ester)	23.87	1.53	C2H7NO_3_S_2_	157
1,2-Benzenedicarboxylic acid, diethyl ester	24.03	1.28	C_12_H_14_O_4_	222
2,3-Dimethoxy-5-methyl-6-dekaisoprenyl-chinon	24.51	0.42	C59H_90_O_4_	862
1-Heptatriacotanol	24.63	0.38	C_37_H_76_O	536
1-(4-Isopropylphenyl)-2-Methylpropyl Acetate	25.65	2.43	C_15_H_22_O_2_	234
1-Heptatriacotanol	26.12	2.08	C_37_H_76_O	536
Tert-Hexadecanethiol	0.89	0.89	C_16_H_34_S	258
Isochiapin B	26.58	0.40	C_19_H_22_O_6_	346
17-Hydroxy-1,17-Dimethylandrostan-3-ONE	26.97	0.29	C_21_H_34_O_2_	318
1,25-Dihydroxyvitamin D3, TMS derivative	27.39	1.26	C_30_H_52_O_3_Si	488
2-Acetyl-3-(2-Cinnamido) Ethyl-7-Methoxyindole	27.88	0.46	C_22_H_22_N_2_O_3_	362
Dotriacontane	28.22	0.39	C_32_H_66_	450
Ethyl iso-allocholate	28.29	0.16	C_26_H_44_O_5_	350
17-Octadecynoic acid	28.46	2.89	C_18_H_32_O_2_	280
Quinindoline	28.97	2.30	C_18_H_14_N_2_	258

^a^Retention time (R.T).

^b^Molecular formula (M.F.).

^c^Molecular weight (M.W).

In line with previous report, quinindoline exhibited numerous pharmaceutical applications, including anticancer, antiarrhythmic, antimalarial, antimicrobial, antioxidant, and astringent activities^34^. In the current study, nonynoic acid was detected in Kei apple extract (Table 2). Nonynoic acids have high fungistatic properties and have shown broad spectrum activity against Gram negative and positive bacteria^35^. Similarly, tert-hexadecanthiol (Table 2) plays an important role in antibacterial and antioxidant activities^36^. The active metabolite of vitamin D (1,25-dihydroxyvitamin D3) was also detected in *D. caffra* fruit extract.

### Antioxidant activity

**Figure 4:**
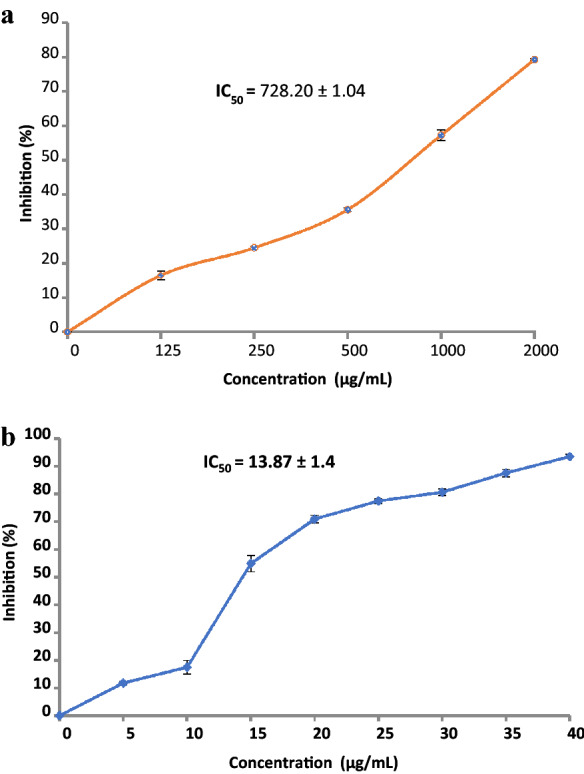
(**a**) Antioxidant activity of *D. caffra* fruit extract. (**b**) Antioxidant activity of ascorbic acid as standard positive.

Most food technology depends on the use of synthetic antioxidants; nevertheless, there is public pressure to alternate these synthetic molecules by searching for an organic alterative. The antioxidant ability of *D. caffra* extract in vitro was carried out to scavenge free radicals 2,2‐di(4‐tert‐octylphenyl)‐1‐picrylhydrazyl radical (DPPH). Given its antioxidant activity (Fig. 4a), the *D. caffra* extract showed antioxidant activity which enhanced with high concentration from 125 µg/mL to 2000 µg/mL, and DPPH scavenging % inhibition was16.46–79.25, respectively. However, the IC_50_ (728.20 ± 1.04 µg/mL) of the *D. caffra* extract was higher than the IC_50_ (13.87 ± 1.4 µg/mL) of ascorbic acid as a synthetic antioxidant (Fig. 4b). These findings are a vital stage in providing scientific evidence of the validation of natural ingredients with promising therapeutic benefits. The antioxidant properties of *D. caffra* extracts are highly connected to their constituents such as quercetin, chlorogenic acid and apigenin that detected by HPLC. These constituents may use to overcome the oxidative and inflammatory stresses. As mentioned previously, antioxidant activity would also be due to attendance of Tert-hexadecanethiol that was detected in *D. caffra* fruits^36^.

A similar trend was reported by Taher et al.^13^, where they noted that fruit of Kei apples had main antioxidants due to the occurrence of high contents of polyphenolic and ascorbic acids. Antioxidant activity of *D. caffra* fruits was attributed to the existence of amino acids^23^.

### Anticancer activity

**Figure 5:**
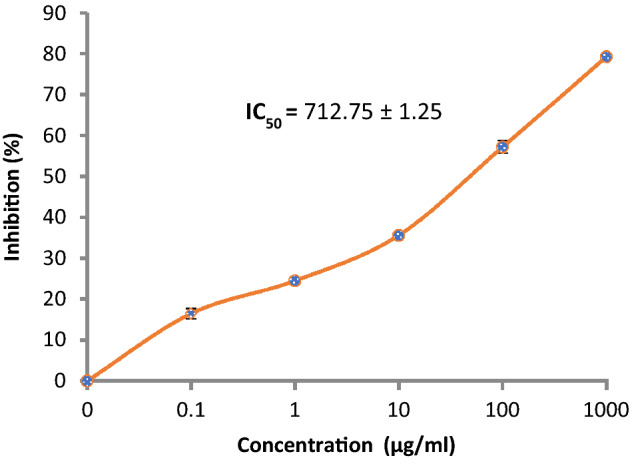
Cytotoxicity of *D. caffra* fruit extract against HepG2.

**Figure 6:**
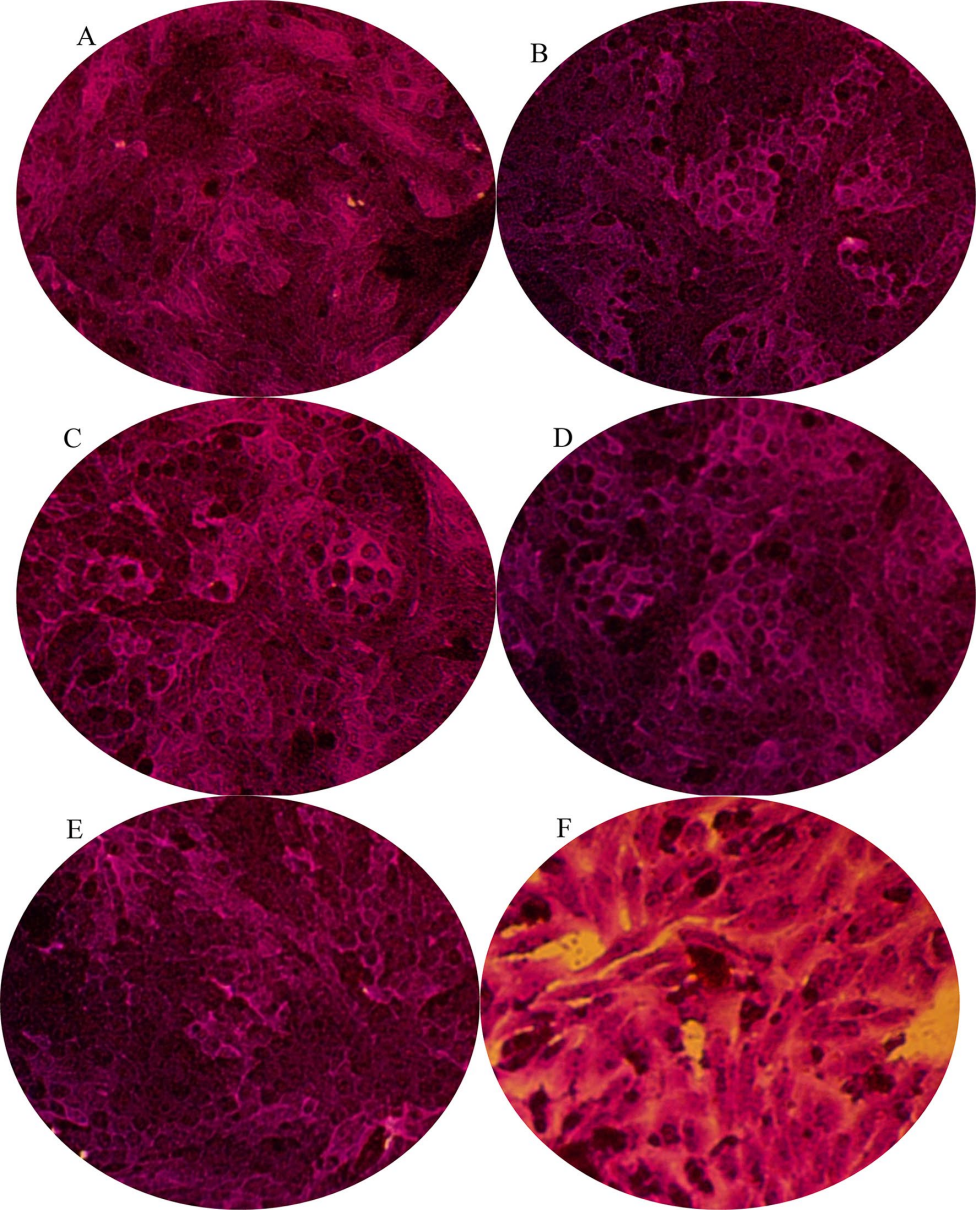
Morphological alteration of treated HepG2 by different concentration of *D. caffra* fruit extract. (**a**) control; (**b**) 0.1 µg; (**c**) 1 µg, (**d**) 10 µg; (**e**) 100 µg; (**f**) 1000 µg.

The lower concentrations of the extract (0.1–10 µg/mL) exhibited very weak anticancer activity against HepG2 cells, evidenced by 90% viability (Fig. 5). *D. caffra* extract at has relatively cytotoxicity that positively associated with concentration, 100 µg/mL lead to 89% viability while 1000 µg/mL, reaching 41.10% viability. The current results are partially in good agreement with earlier literatures, showing the in vitro anticancer activity of *D. caffra* branches against colon (HCT-116), breast (MCF-7), lung (A-549) and hepatocellular (HepG2) carcinomas^37^. HepG2 cell lines exposed to the extract showed morphological changes in a concentration-dependent manner. HepG2 cells exposed to 0.1-10 μg/mL exhibited no morphological changes while at 100 μg/mL, cells loss their adhesion capacity. At high concentration (1000 μg/mL), in addition to loss of cell adhesion capability, most of the cells shrank and abnormal morphology (the presence of rich cytoplasmic vacuoles) was observed, (Fig. 6).

### Antibacterial and antifungal activities

Extracts of Kei apple fruit showed potential antibacterial activity with potency against gram-negative bacteria (*E. coli* and *P. vulgaris*) compared to gram-positive species (*S. aureus* and *B. subtilis*) (Table 3). The antimicrobial activity was compared against gentamicin, where the extract produced a large zone of inhibition, similar to gentamicin against *Proteus vulgaris*. The world health organization listed gram-negative bacteria, including *E. coli* at the top of antibiotic-resistant organisms, which required immediate action. The results of the current study suggest the potential use of Kei apple fruit extract as a narrow spectrum antimicrobial agent for gram-negative bacteria, although more bacteria and further risk assessment is needed. Moderate antibacterial potential toward *S. aureus, Streptococcus faecalis, Klebsiella pneumonia, Pseudomonas aeruginosa* and *Streptococcus pyogenes* was documented in vitro^38^. On the other hand, the antifungal activity test showed weak activity against the unicellular fungus *Candida albicans* ATCC 10231 and showed no activity against the filamentous fungus *Aspergillus fumigatus* (Table 3). Although few scientific studies have focused on the antimicrobial activity of natural extracts from *D. caffra*, our study indicates the possible use of extracts as antimicrobial agents.

**Table 3:** Antimicrobial activity of *D. caffra* fruit extract against clinically important pathogens including fungi, Gram positive and negative bacteria.

Tested microorganisms	Inhibition zone (mm)	
	Extract (100 µl)	Control^a^
**Fungi**		
*A. fumigatus* (RCMB 002008)	0.0	17
*C. albicans* ATCC 10231	10	20
**Gr+ve bacteria**		
*S. aureus* ATCC 25923	15	24
*B. subtilis* NRRL B-543	13	26
**Gr−ve bacteria**		
*E. coli* ATCC 25922	22	30
*P. vulgaris* ATCC 13315	24	25

^a^Positive control Ketoconazole for fungi and Gentamycin for bacteria. The test was done using the diffusion agar technique, well diameter: 6.0 mm (100 µl was tested).

### Antiviral activity

**Figure 7:**
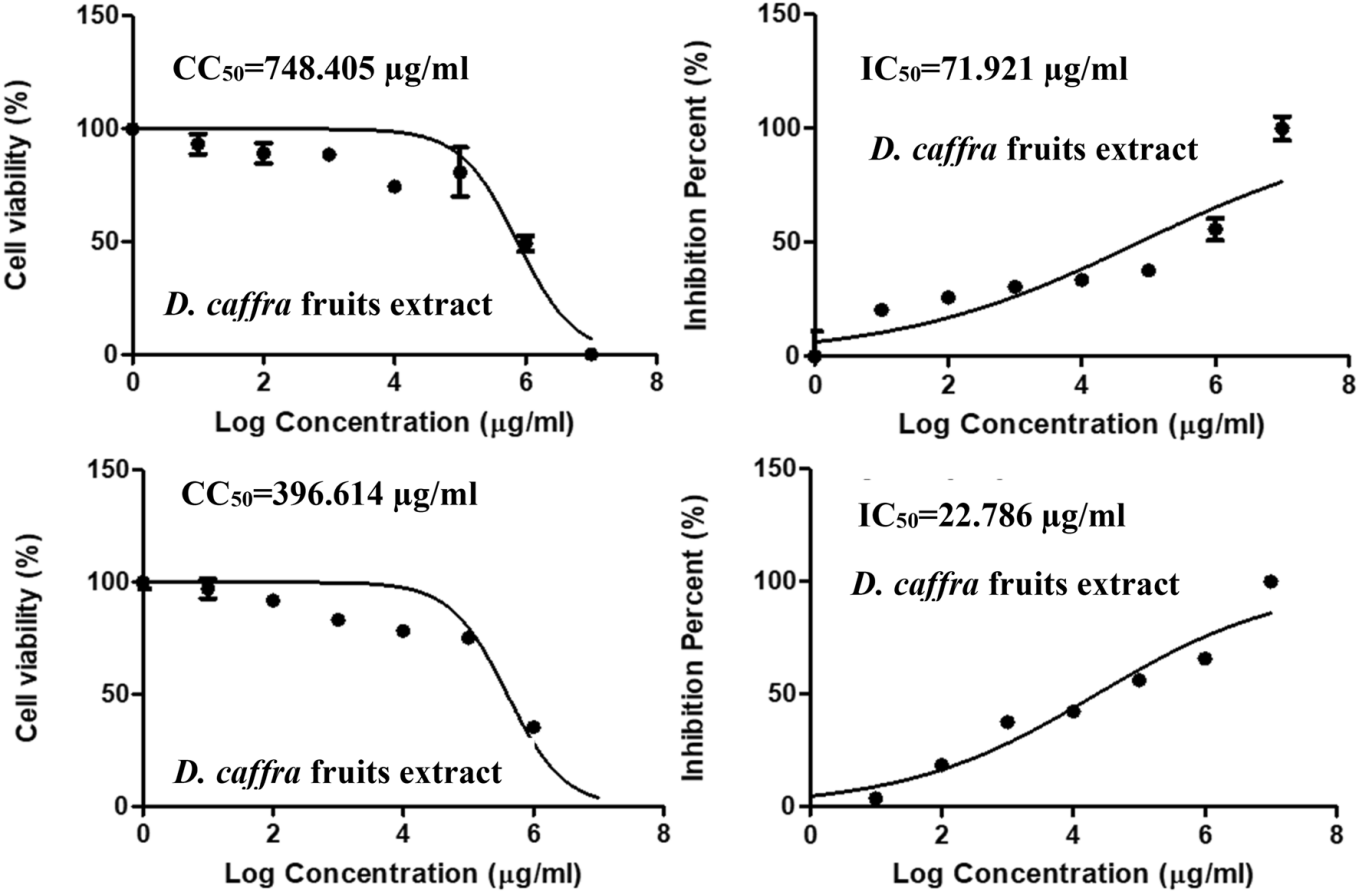
Effect of *D. caffra* fruits extract on Coronavirus 229E with Acyclovir as a positive control.

**Figure 8:**
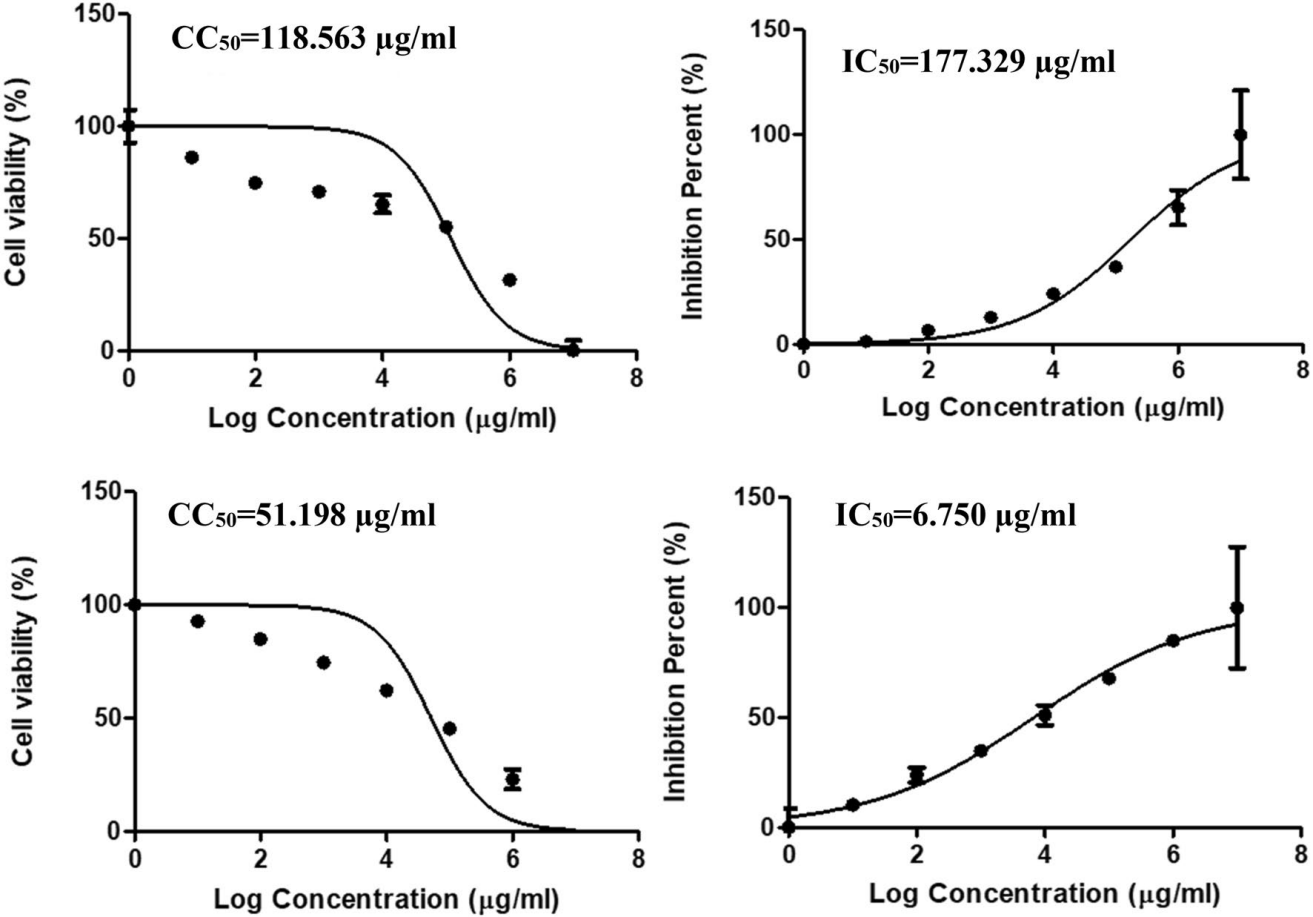
Effect of *D. caffra* fruits extract on human H3N2 with Acyclovir as a positive control.

The cytotoxic concentration (CC_50_) of the extract was 748 µg/mL, and the IC_50_ was 71.92 µg/mL and selective index was 10.4, (Table 4 and Fig. 7). This indicating mild antiviral activity against human coronavirus and further experiments can be done to improve its anti-coronaviruses activity. It is commonly known that when IC_50_ concentration is below CC_50_ concentration, this would mean that the virus will be killed before causing damage to host cells and they will not suffer any adverse effects if treated with the extracts. On the other hand, *D. caffra* extract showed no antiviral activity against human H3N2, with an IC_50_ (177.33 µg/mL) greater than the CC_50_ (118.56 µg/mL), in addition to a selective index of 0.67 (Table 4 and Fig. 8). There are no reports of *D. caffra* fruits against viruses, although some fruit extract have demonstrated antiviral activity^39^. Heyman et al.^40^ mentioned that satisfactory evidence about the viricidal potential of natural products has been documented over the years and has still been discovered in numerous species of plants^40^. The HPLC analysis revealed that the chlorogenic acid was the main detected ingredient of the *D. caffra* fruit extract, and the useful activity of these acids was reported against herpes simplex viruses (HSV), but not against human immunodeficiency virus (HIV)^41^.

**Table 4:** Antiviral activity of *D. caffra* fruits extract against Human coronavirus 229E and human H3N2.

Treatment extract	Virus	**CC**_**50**_ (µg/ml)	**IC**_**50**_ (µg/ml)	*SI
*D. caffra* fruits	Human coronavirus 229E	748.41	71.92	10.4
Acyclovir		396.61	22.79	17.4
*D. caffra* fruits	Human H3N2	118.56	177.33	0.67
Acyclovir		51.20	6.75	7.58

### Molecular docking of chlorogenic acid with HCoV-229E and *Proteus vulgaris*

**Figure 9:**
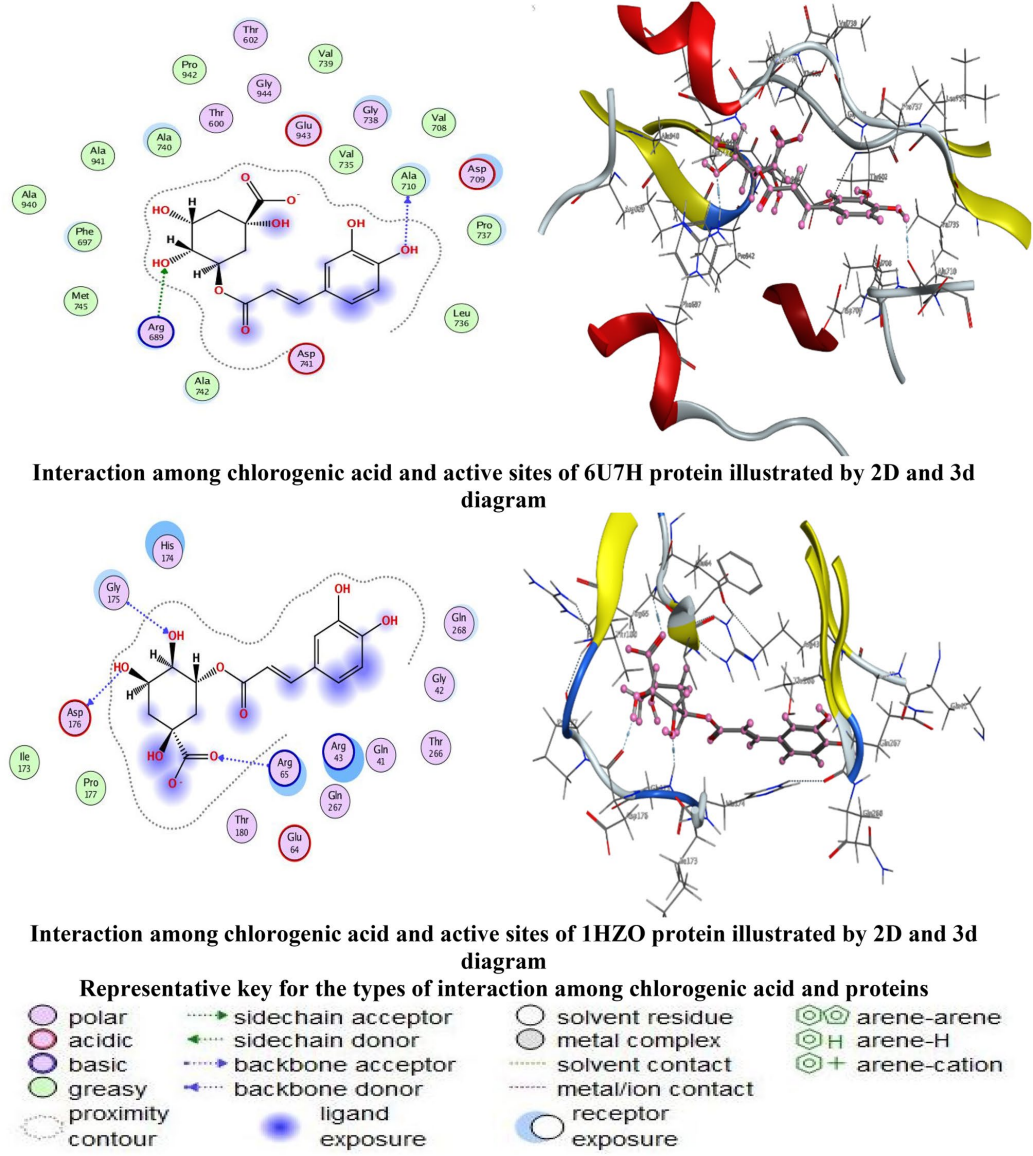
Docking interactions of chlorogenic acid and active sites of 6U7H and 1HZO protein proteins.

Chlorogenic acid was the main detected constituent in *D. caffra* fruit extract as well as its biological activities was selected to study the molecular docking with HCoV-229E and *Proteus vulgaris*. Molecular docking has been applied to chlorogenic acid with Cryo-EM structure of the HCoV-229E spike glycoprotein (6U7H) and structure of class A cephalosporinase from *Proteus vulgaris* K1 (1HZO) as showed (Fig. 9), which were chosen from the literature, to investigate the binding mode and the conformation structure that contributes to the interaction between the proteins and the ligand as. Molecular docking is a kind of bioinformatic modelling which involves the interaction of two or more molecules to give the stable adduct. Depending upon binding properties of ligand and target^42^. Molecular docking generates different possible adduct structures that are ranked and grouped together using scoring function in the software. As well as in the mechanistic study by placing a molecule (ligand) into the preferred binding site of the target specific region of the DNA/protein (receptor) mainly in a non-covalent fashion to form a stable complex of potential efficacy and more specificity^43^. The information obtained from the docking technique can be used to suggest the binding energy, free energy and stability of ligand. At present, docking technique is utilized to predict the tentative binding parameters of ligand-receptor complex beforehand. The hydrogen bonds formed between the receptors and the chlorogenic acid were used to rank the binding affinity and were presented as the free binding energy (S, kcal/mol). Chlorogenic acid showed the highest docking score of − 6.9689 kcal/mol with (6U7H) which is higher than that of the other protein (1HZO) with − 6.5072 kcal/mol. Tables 5 and 6 reveal the following results: chlorogenic acid have a higher negative score of free binding energy with both proteins (6U7H) and (1HZO), indicating the applicability of chlorogenic acid by encouraging antivirus and antibacterial drugs that could help medicinal chemists and pharmaceuticals further design and synthesize more effective drug candidates. The HCoV-229E protein (6U7H) interacted via amino acid pocket molecules with O 41and O 17 by donating their H atoms or accepting H atoms through O ALA 710 and NH2 ARG 689 receptors. The interaction with *Proteus vulgaris* protein (1HZO) formed one hydrogen donor atom between O19 in ligand and ASP 176 amino acid receptor, in addition to the two hydrogen acceptor interaction between O17 and O23 atoms in ligand and GLY 175 and ARG65 amino acids receptors, respectively. The DFT-optimized structures of the chlorogenic acid were used to generate the best five binding poses with flexible molecules rotation as shown in Tables 7 and 8.

**Table 5:** Chlorogenic acid interaction with 6U7H protein.

Chlorogenic acid	Receptor	Interaction	Distance	E (kcal/mol)
O 41	O ALA 710 (A)	H-donor	3.01	− 1.3
O 17	NH2 ARG 689 (A)	H-acceptor	2.99	− 2.9

**Table 6:** Chlorogenic acid interaction with 1HZO protein.

Chlorogenic acid	Receptor	Interaction	Distance	E (kcal/mol)
O 19	O ASP 176 (A)	H-donor	2.91	− 0.6
O 17	N GLY 175 (A)	H-acceptor	2.90	− 2.0
O 23	N ARG 65 (A)	H-acceptor	3.00	− 2.9

**Table 7:** Docking score and energies of chlorogenic acid with 6U7H receptors.

Mol	mseq	S	rmsd_refne	E_conf	E_place	E_score1	E_refne	E_score2
	1	− 6.9689	1.3119	− 3.7118	− 102.5658	− 11.4025	− 41.9138	− 6.9689
	1	− 6.8187	2.3957	− 10.5703	− 83.9046	− 11.9740	− 41.2830	− 6.8187
	1	− 6.7687	1.7142	− 13.5838	− 82.7499	− 11.8091	− 36.1343	− 6.7687
	1	− 6.6799	1.8245	− 11.4598	− 78.5136	− 11.8723	− 37.9954	− 6.6799
	1	− 6.6611	1.2779	− 14.0470	− 86.4123	− 11.6929	− 39.1225	− 6.6611

**Table 8:** Docking score and energies of chlorogenic acid with 1HZO receptors.

Mol	mseq	S	rmsd_refne	E_conf	E_place	E_score1	E_refne	E_score2
	1	− 6.5072	1.4780	− 14.4617	− 91.6791	− 11.7837	− 36.4847	− 6.5072
	1	− 6.4053	1.0339	− 14.1381	− 94.8616	− 11.4590	− 33.3535	− 6.4053
	1	− 6.4011	1.5723	− 14.1350	− 65.5731	− 13.4136	− 33.3605	− 6.4011
	1	− 6.3808	1.7736	− 8.9460	− 97.8223	− 12.4328	− 31.6119	− 6.3808
	1	− 6.3475	1.3461	− 13.5739	− 81.7388	− 11.5236	− 31.7171	− 6.3475

## Conclusion

In the present work, we reported the identification of various phytoconstituents in Kei apple fruit by using both GC–MS and HPLC techniques. Chlorogenic acid and catechin were the main identified compounds together with furyl hydroxymethyl ketone, pogostole, and 2-Butenedioic acid (Z)-, monomethyl ester. The tested juice extract was able to inhibit the growth of four bacterial strains (*S. aureus*, *B. subtilis*, *E. coli*, *P. vulgaris*) and one yeast (*C. albicans*). Moreover, Kei apple methanolic extract was active against human coronavirus 229E and exhibited antioxidant and anticancer activities. These findings highlighted the beneficial use of *D. caffra* fruit extract as source of bioactive compounds to be used as an alternative therapeutically agent. Energy scores of the molecular docking of chlorogenic acid with HCoV-229E spike glycoprotein (6U7H) and A cephalosporinase of *Proteus vulgaris* K1 (1HZO) receptor results in excellent synchronization with the experimental findings.

## Data Availability

All data that support the findings of this study are available within the article.

## Abbreviations

RCMB: Al-Azhar University at Regional center for mycology and biotechnology
CC_50_: Cytotoxic concentration
GC/MS: Gas chromatography/mass spectrometry
HPLC: High-performance liquid chromatography
CPE: Cytopathogenic effect
NIST: National Institute of Standards and Technology
DMEM: Dulbecco’s modified Eagle’s medium
Gr + ve: Gram positive
Gr-ve: Gram negative
FBS: Fetal bovine serum
TCA: Trichloroacetic acid
YEPD: Yeast extract peptone dextrose
IC_50_: Inhibitory concentration 50%
CCID_50_: 50% Cell culture infective dose
SI: Selective index
PBS: Phosphate-buffered saline
ODCC: Control optical density of cell
ODVC: Control optical density of virus
ODT: Test optical density
DPPH: 2-Diphenyl-1-picryl-hydrazyl-hydrate
